# Neural field model to reconcile structure with function in primary visual cortex

**DOI:** 10.1371/journal.pcbi.1005821

**Published:** 2017-10-24

**Authors:** James Rankin, Frédéric Chavane

**Affiliations:** 1 Department of Mathematics, University of Exeter, Exeter, United Kingdom; 2 Center for Neural Science, New York University, New York, New York, United States of America; 3 Institut de Neurosciences de la Timone, CNRS & Aix-Marseille Université, Faculté de Médecine, Marseille, France; Hamburg University, GERMANY

## Abstract

Voltage-sensitive dye imaging experiments in primary visual cortex (V1) have shown that local, oriented visual stimuli elicit stable orientation-selective activation within the stimulus retinotopic footprint. The cortical activation dynamically extends far beyond the retinotopic footprint, but the peripheral spread stays non-selective—a surprising finding given a number of anatomo-functional studies showing the orientation specificity of long-range connections. Here we use a computational model to investigate this apparent discrepancy by studying the expected population response using known published anatomical constraints. The dynamics of input-driven localized states were simulated in a planar neural field model with multiple sub-populations encoding orientation. The realistic connectivity profile has parameters controlling the clustering of long-range connections and their orientation bias. We found substantial overlap between the anatomically relevant parameter range and a steep decay in orientation selective activation that is consistent with the imaging experiments. In this way our study reconciles the reported orientation bias of long-range connections with the functional expression of orientation selective neural activity. Our results demonstrate this sharp decay is contingent on three factors, that long-range connections are sufficiently diffuse, that the orientation bias of these connections is in an intermediate range (consistent with anatomy) and that excitation is sufficiently balanced by inhibition. Conversely, our modelling results predict that, for reduced inhibition strength, spurious orientation selective activation could be generated through long-range lateral connections. Furthermore, if the orientation bias of lateral connections is very strong, or if inhibition is particularly weak, the network operates close to an instability leading to unbounded cortical activation.

## Author Summary

Optical imaging techniques can reveal the dynamical patterns of cortical activation that encode low-level visual features like position and orientation, which are shaped by both feed-forward projections, recurrent and long-range intra-cortical connections. Anatomical studies have characterized intra-cortical connections, however, it is non-trivial to predict from this data how evoked activity might spread across cortex. Indeed, there remains an apparent conflict between the reported orientation bias of cortical connections, and imaging studies on the propagation of cortical activity. Our study reconciles structure (anatomy) with function (evoked activity) using a dynamic neural field model that predicts the dynamics of cortical activation in a setting both inspired by and parametrically matched to the available anatomical data.

## Introduction

Horizontal connections link cells separated within the same cortical area, over a distance of a few millimeters (mm) covering several iso-functional columns [1, 2] and spatially distributed into regular clusters [3–5]. In V1, the lattice-like pattern of connectivity is reminiscent of the spatial regularity observed in orientation maps and has therefore been proposed to link neurons with similar preferred orientation [6]. Functional mapping combined with retrograde labeling [7] has shown that pyramidal cell horizontal axons have a bias to preferentially connect iso-orientation loci (in cat [8, 9], tree shew [10] and monkey [11]). However, this result depends on cell type and location since neurons in layer 4 (L4) [12], pinwheel centers ([13] but see [14, 15] since this result may depend critically on the distance to the pinwheel center) and inhibitory cells [16, 17] connect without orientation bias. As a consequence of this cellular heterogeneity, it is not trivial to predict the selectivity of stimulus-driven horizontal activation.

**Fig 1:**
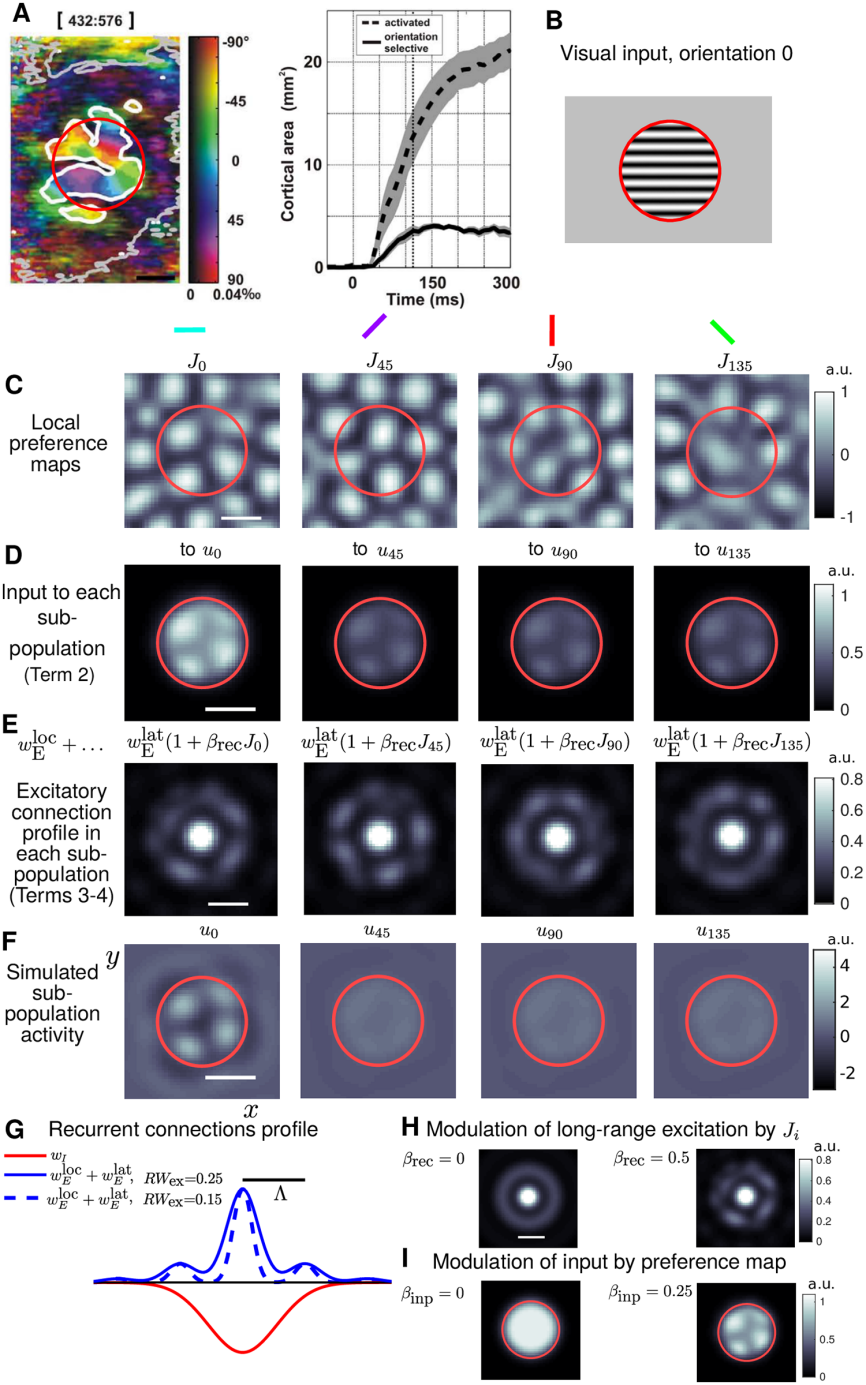
Model schematic and main parameters. **A:** Adapted from [24]. VSD-imaged responses (left) from cat area 17 (V1) showing orientation preference (color) and degree of orientation selectivity (color intensity), calculated using vector averaging from four different presentations of stimuli like B. Region inside the grey contour (extending to the edges of the imaging window) showed significant general activation. A smaller sub-region inside the white contour is significantly orientation selective. Time-courses (right), averaged from 9 cortical locations, show the orientation-selective area (solid) saturates after around 120 ms whilst the general activation (dashed) spreads further and has not saturated by 300 ms. **B:** Localized grating in the visual field with orientation 0°. The edge of the circular aperture is red for visualisation. **C:** Local portion of the preference map components *J*_0_, *J*_45_, *J*_90_ and *J*_135_, each associated with a different orientation, see Fig 2 for further details. Scale bars (black or white) are equal to *Λ* (hypercolumn separation) throughout the manuscript. **D:** Model inputs to each sub-population for a stimulus like B, where the orientation is encoded by a stronger input weighting for the input to *u*_0_, and spatial modulation by *J*_0_ (see I). Red circle is the FFF of the input. **E:** Excitatory component of recurrent connections within each sub-population, the long-range connections are modulated by the correspond map *J*_*i*_. **F:** Four sub-populations, *u*_0_, *u*_45_, *u*_90_ and *u*_135_, are each associated with a different orientation. A stimulus with a specific orientation will elicit a patterned activation in the corresponding sub-population (here *u*_0_) and sub-threshold responses in the other populations (*u*_45_, *u*_90_ and *u*_135_). The multi-spot patterns (see *u*_0_) are encoded by the recurrent connectivity profile (panel G). Modulation of either the inputs (D) or long-range recurrent connections (E) by a preference map (C) fixes the location of the spots for a given orientation. **G–I:** Effect of parameters *RW*_ex_, *β*_rec_ and *β*_inp_. **G:** Cross-section of the radial connectivity profiles for excitation and inhibition. There are regular peaks in excitation each *Λ*-distance (black bar) from the origin. A parameter *RW*_ex_ controls the width of these peaks (broader for larger values). **H:** In each sub-population *u*_*i*_ the long-range connections (rings at *Λ*, 2*Λ*) are modulated by the corresponding orientation preference map when *β*_rec_ > 0. **I:** The parameter *β*_inp_ controls the modulation strength of the inputs by the orientation preference map.

More information was recently gained from population measures of orientation selectivity at mesoscopic scales. Techniques such as optical imaging has led to the description of cartographic organization of many cortical structures [18, 19]. The development of voltage-sensitive dye [20] has further allowed for investigation of dynamic features and computations arising within these maps, see reviews in [21] and [22]. In [23] voltage sensitive dye imaging (VSDI) was used to study the retinotopic activation with localized oriented inputs in cat V1. A characteristic plateau of activity, coinciding with the retinotopic extent of the stimulus, was independent of stimulus orientation. Within the plateau several peaks of activation would appear, with location strongly dependent on orientation. [24] further explored orientation selectivity outside the retinotopic activation using localized stimuli. Over several hundred milliseconds activation gradually propagated outwards, extending several mm beyond the feedforward footprint (FFF). The dynamic and the spatial range of this activation beyond the FFF is presumably generated by long-range excitatory connections in L2/3 of V1. Note that, although less plausible because their spatial and temporal properties are not in the appropriate range (see discussion in [25] and [24]), alternative circuits such as intra-thalamic, thalamo-cortical divergence and feedback loops cannot be entirely dismissed (but see [26]). Interestingly, only a local component of this activation, circumscribed within the FFF, was found to be orientation selective (Fig 1A), at first glance a surprising finding given the numerous studies showing an iso-orientation bias for long-range connections. However, as discussed in [24] and introduced above, this result may be expected from the anatomy known from studies already published at that time. First, because the iso-orientation connection bias is small and has been quantified only for short horizontal distances (mostly <1–1.5 mm, a distance for which a similar small bias is also reported by [24]). Second, because it can be seen in the few existing intracellular labeling studies that the iso-orientation bias tends to decrease with distance [17, 14]. Thus, the results from [24], although in contradiction with a strict “like-to-like connectivity” principle, call for modification depending on cortical distance: from a like-to-like bias in functional connectivity at short range toward no bias at long range. Importantly, two follow-up studies consolidated these findings with an optogenetics functional confirmation ([27], see also [28, 29]) and a timely anatomical clarification using quantitative and statistical analysis of intracellular labeled neurons [15].

The aim of this modelling study is to explore the relationship between lateral connectivity properties (structure) and the way activity spreads across cortex as evoked by localized, oriented stimuli (function). It remains to reconcile the studies of iso-orientation bias of anatomical connectivity in V1 [7, 9, 14] and the properties of cortical activation observed in other experiments [24, 27, 15]. To achieve this, we have developed a dynamical model to investigate stimulus driven activity in 2D cortical space with a discrete representation of orientation. The neural field equation gives a coarse-grained description of average membrane potential on a continuous domain [30, 31]. This framework has been widely used to model visual cortex (e.g. [32–35]) and cortical dynamics in general (see reviews [36] and [37]). This level of description is well-suited for comparison with VSDI [38–40]. The model’s design allows for connectivity properties such as the spatial profile of excitation and inhibition to be investigated. Model parameters were constrained to be consistent with the level of orientation bias reported in [14]. Our study shows that realistic cortical connectivity schemas [14, 15] govern a spatio-temporal dynamics of cortical activation in accordance with the observed functional dynamics [24, 27]. Our approach allowed us to further elaborate on the non-trivial link between anatomical constraints and predictions of population level activation patterns. To probe the structure-function link, we used our model to make experimentally tractable predictions on the specific role of excitatory-inhibitory balance.

## Methods

### Outline of the model

Our neural field equation model [30, 31] gives a mesoscopic, continuous description of neural activity on a 2D plane (*x*, *y*) parallel with the cortical surface in layer 2/3 of V1. Neural activity is represented by an average membrane potential *u*_*i*_(*x*, *y*, *t*) evolving with time *t* in four sub-populations, each associated with a different orientation *i* = {0°, 45°, 90°, 135°} (the ° notation will be dropped in the remainder of the manuscript). The decomposition into sub-populations is a convenient abstraction for modelling purposes; these sub-population outputs *u*_*i*_(*x*, *y*, *t*) will be combined into activity variables that can be compared with experimental data in *Conversion of model output to VSD-like signal*. The following integro-differential equation describes the dynamics of the *u*_*i*_ population:

$$\tau \frac{\partial }{\partial t}u_{i}\left(x,y,t\right)=-\underset{j}{\sum }\rho_{j}u_{j}\left(x,y,t\right)$$ (1)

$$+\;\underset{j}{\sum }k_{j}I_{j}\left(x,y\right)\left(1+\beta_{\text{inp}}J_{j}\left(x,y\right)\right)$$ (2)

$$+\;S\left(u_{i}\left(x,y,t\right)\right)\;\overset{\left(x,y\right)}{⋆}\left[g_{\text{ex}}w_{\text{E}}^{\text{loc}}\left(x,y\right)-g_{\text{in}}w_{\text{I}}\left(x,y\right)\right]$$ (3)

$$+\;\left(1+\beta_{\text{rec}}J_{i}\left(x,y\right)\right)S\left(u_{i}\left(x,y,t\right)\right)\overset{\left(x,y\right)}{⋆}g_{\text{ex}}w_{\text{E}}^{\text{lat}}\left(x,y\right).$$ (4)

The cortical timescale is *τ* = 10 ms, and the rate of change of *u*_*i*_ is proportional to the right hand side of this equation with the following terms

- (1) Decay of the population activity to resting potential
- (2) Stimulus driven input with feedforward footprint *I*, modulated by the orientation map *J*
- (3) Non-selective intra-cortical interactions gated by sigmoidal threshold function *S*
- (4) Orientation-selective interactions, modulated by the orientation map *J*

Term (1) describes a decay back to an resting potential of *u*_*i*_ = 0 (the membrane potential has arbitrary units). Within the sub-population the decay term has strength *ρ*_*i*=*j*_ = 1 (an arbitrary choice for this first constant). Between sub-populations the decay term has strength *ρ*_*i*≠*j*_ = 0.1 (local, linear cross inhibition). For *ρ* > 0.2 this cross inhibition can generate undesired above-threshold activation in non-stimulated sub-populations, therefore, a value 50% below this was selected. Term (2) describes localised circular inputs with one of four orientations (e.g. Fig 1B). For an input with say orientation 0, *I*_0_ is active and *I*_45_, *I*_90_ and *I*_135_ are zero. The input weighting for a sub-population’s associated orientation *k*_*i*=*j*_ = *k*_1_ is larger than the weighting for other orientations *k*_*i*≠*j*_ = *k*_2_, with *k*_1_ = 2*k*_2_. Inputs are modulated by the orientation map *J*_*i*_ with strength *β*_inp_ = 0.25 (Fig 1D and 1I). For *β*_inp_ > 0.2 the spatial phase of multi-bump patterns of activity will match the orientation preference map as required, increasing it further has little effect. Term (3) describes non-selective lateral connections within the sub-population *u*_*i*_ via a convolution ⋆ over (*x*, *y*) (spatial integrals, see (6)) with the components of the spatial connectivity profile. This profile is radially symmetric and describes the average connectivity at any point in cortex. It is broken down into local excitatory $w_{E}^{\text{loc}}$ and inhibitory *w*_I_ components, see Fig 1G and *Details and equations for connectivity profile*. In the convolutions *u*_*i*_ is processed through the sigmoidal threshold function *S*, which transforms membrane potential into a normalized firing rate. Any locations above threshold will influence their neighbors through lateral connections. Weighting constants *g*_ex_ and *g*_in_ are determined by theoretical constraints, see *Details and equations for connectivity profile* and (17). Term (4) describes orientation-selective excitatory connections as modulated by the preference map *J*_*i*_ with strength *β*_rec_.

An example of the simulated sub-population activity is shown in Fig 1F for an input with orientation 0. The main parameters of interest in this study are *RW*_ex_, which modifies the spatial width of excitatory peaks (Fig 1G) and *β*_rec_ (taking values between 0 and 1), which controls the amplitude modulation of long-range excitation by the orientation preference map (Fig 1H). We will also study a parameter *C* controlling the strength of inhibition (equivalently the balance between excitation and inhibition) as defined by (16) in *Details and equations for connectivity profile*.

The firing rate (or threshold) function is given by a sigmoid

$$\begin{array}{c} S\left(u\right)=\frac{1}{1+e^{-\mu u+\theta }}-\frac{1}{1+e^{\theta }},\;\mu ,\theta >0, \end{array}$$ (5)

where *μ* = 2.3 is a slope parameter and *θ* = 5.6 the threshold. Input strengths *k*_1_ = 2.8 and *k*_2_ = 1.4 are set such that inputs to the stimulated sub-population are above threshold in (5) *μk*_1_ > *θ*, whilst inputs to other sub-populations are below threshold *μk*_2_ < *θ*. The particular form of *S* is chosen such that *u*_*i*_ = 0 (all populations at resting potential) is a always a solution to the model equations and values of *μ* and *θ* are chosen such that this is the only stable solution with no inputs [41]. The spatial convolution terms in (3)–(4) are expressed as integrals, e.g. Term (4) can be computed as one integral

$$\begin{array}{l} w_{\text{E}}^{\text{lat}}\left(x,y\right)\overset{\left(x,y\right)}{⋆}\left[S\left(u_{i}\left(x,y,t\right)\right)\left(1+\beta_{\text{rec}}J_{i}\left(x,y\right)\right)\right]=\\ \;\;\;\;\;\;\;\;\;\;\;\;\;\;\;\;\;\;+\int_{x,y}\;w_{\text{E}}^{\text{lat}}\left(x-x^{\prime },y-y^{\prime }\right)\;\left[S\left(u_{i}\left(x^{\prime },y^{\prime },t\right)\right)\;\left(1+\beta_{\text{rec}}J_{i}\left(x^{\prime },y^{\prime }\right)\right)\right]\;dx^{\prime }dy^{\prime }, \end{array}$$ (6)

with dummy variables *x*′ and *y*′. In this case the modulation by *J*_*i*_ is introduced whilst still preserving the convolutional structure, allowing for the numerical methods described in [41], exploiting Fast Fourier Transforms to be applied. The radial inputs *I*_*i*_(*r*), $r=\sqrt{x^{2}+y^{2}}$, as plotted in Fig 1I(left), are given by

$$\begin{array}{c} I_{i}\left(r\right)=\{\begin{array}{cc} 1,\; & r<0.7\Lambda \\ h(r,0.7\Lambda ,0.3\Lambda )\; & r\geq 0.7\Lambda . \end{array} \end{array}$$ (7)

where *h* is the radially shifted Gaussian ring given below in (10). The extent of *I*_*i*_ and its decay at the stimulus border were chosen to match cortical point spread functions measured in [24]. In simulations the input amplitude ramps up linearly from 0 to full amplitude in the interval *t* ∈ [20, 120] ms, see [40].

For each sub-population a finite differencing scheme for the domain [−*L*, *L*] × [−*L*, *L*] with *L* = 30 and *N* = 128 evenly distributed gridpoints in each spatial direction is used, as described in [41]. Model simulations were run on a domain much larger (2*L* = 60, relative to *Λ* = 2*π*) than the localized patterns of activation studied here, justifying the use of periodic boundary conditions. A standard Runge-Kutta time stepper in Matlab was used for model simulations with default tolerances. The source code for the full implementation of the model has been made available in the Supplemental Information (S1 Code).

### Details and equations for connectivity profile

Earlier theoretical works characterized connectivity constraints that lead to stable localized patterns of activation in 1D [42, 43] and in 2D [44, 45]. A common choice of connectivity function is a so-called Mexican hat (e.g. a difference of Gaussians) featuring a broader footprint for inhibition than for excitation [36, 37]. [41] suggested a role for longer-range excitation serving to stabilize larger patterns of activation and equating the separation between peaks in excitation with *Λ* (hypercolumn separation, [46]) The formulation presented here is further inspired by the way [14] quantified their results so that we can link model parameters with their quanitification or orientation bias for long-range connections (see next section). The model’s connectivity profile is broken down into local excitatory $w_{E}^{\text{loc}}$ (a local Gaussian bump), lateral excitatory $w_{E}^{\text{lat}}$ (Gaussian rings centered at *Λ* and 2*Λ*) and inhibitory *w*_I_ (a broad local Gaussian bump) components, which were plotted in Fig 1G. The use of Gaussian rings was inspired by [16] (see their Fig 16), noting the important features that excitatory connections 1) drop in number at a range *Λ*/2, 2) have a peak at a range *Λ* and 3) can extend several mm across cortex. The following details give full definitions of these components and their relative scaling with particular attention to the global balance between excitation and inhibition, which will be controlled by a parameter *C*. When *C* = 0 excitation and inhibition are balanced (the area under the 2D radial versions of the blue and red curves in Fig 1G would be equal). In this study *C* is taken to be negative (net inhibition) whilst remaining close to the balanced condition. We note that when *C* is larger (or even positive) localized patterns of activity are more likely to destabilize and spread across cortex [41], which is investigated in *Reduced inhibition leads to orientation selective activation outside stimulus footprint*. The length scale *Λ* is the mean hypercolumn separation.

Radially symmetric functions for the connectivity components are defined in terms of a radial coordinate $r=\sqrt{x^{2}+y^{2}}$. We define a 2D Gaussian function with spatial decay rate *σ*:

$$g\left(r,\sigma \right)=\frac{1}{2\pi \sigma^{2}}e^{\left(-\frac{r^{2}}{2\sigma^{2}}\right)},$$ (8)

where the pre-factor $\frac{1}{2\pi \sigma^{2}}$ normalizes the area. The number of inhibitory connection, based on a diverse class of inhibitory neurons [16], is assumed to have a Gaussian decay with distance from the origin:

$$\begin{array}{c} w_{I}\left(r\right)=g\left(r,RW_{\text{in}}\right), \end{array}$$ (9)

with *RW*_in_ = 0.55*Λ* (a cross-section of this function is plotted red in Fig 1G). This value gives the qualitative feature that there is more excitation than inhibition at ranges *Λ* and above. The results are not contingent on the exact value chosen, but varying *RW*_in_ has a similar effect to varying *C* (inhibition strength, defined below), which is investigated in *Reduced inhibition leads to orientation selective activation outside stimulus footprint*. Assuming that there are peaks in the number of excitatory connections ever *Λ*-distance from the origin up to 2*Λ*, there are rings of excitation at distances {0, *Λ*, 2*Λ*}. We assume that the amplitude of the peaks centered at these distances decay within an exponential envelope

$$\begin{array}{c} \chi \left(r,\zeta \right)=e^{-\frac{r}{\zeta }}, \end{array}$$

where *ζ* = 0.625*Λ*. The exact value chosen is not critical, varying *ζ* by ±20% does not significantly affect the results. We define a radially shifted 2D Gaussian

$$\begin{array}{c} \begin{array}{c} h\left(r,r_{0},\sigma \right)=e^{\left(-\frac{{\left(r-r_{0}\right)}^{2}}{2\sigma^{2}}\right)}, \end{array} \end{array}$$ (10)

which describes a ring that is maximal at a radius *r* = *r*_0_ and decays away with spatial scale *σ* (note that $\frac{1}{2\pi \sigma^{2}}h\left(r,0,RW_{\text{ex}}\right)=g\left(r,RW_{\text{ex}}\right)$). The local excitatory component (a Gaussian bump centered at 0) is given by

$$\begin{array}{c} w_{E}^{\text{loc}}\left(r\right)=h\left(r,0,RW_{\text{ex}}\right), \end{array}$$ (11)

where *RW*_ex_ is a free parameter. For *RW*_ex_ < 0.1 the number of excitatory connections at *Λ*/2 would drop to 0, which is not realistic. Further *RW*_ex_ should be less than *RW*_in_; for our parameter exploration, we therefore consider a smaller range [0.1,0.4] based on the anatomical constraints introduced later. The long-range excitatory component (Gaussian rings centered at *Λ* and 2*Λ*) is given by

$$\begin{array}{c} w_{E}^{\text{lat}}\left(r\right)=\chi \left(\Lambda ,\zeta \right)h\left(r,\Lambda ,RW_{\text{ex}}\right)+\chi \left(2\Lambda ,\zeta \right)h\left(r,2\Lambda ,RW_{\text{ex}}\right). \end{array}$$ (12)

The overall profile of excitation $w_{E}^{\text{loc}}+w_{E}^{\text{lat}}$ is plotted in Fig 1G. Noting the following analytic expression for the zero-mode of the Fourier transform of (10)

$$\begin{array}{c} H\left(0,r_{0},\sigma \right)=2\pi \sigma^{2}e^{-\frac{r_{0}^{2}}{2\sigma^{2}}}+\pi \sigma r_{0}\sqrt{2\pi }(1+\mathrm{erf}\frac{r_{0}}{\sqrt{2}\sigma }), \end{array}$$ (13)

where erf is the standard error function, we write the normalisation pre-factor for the combined excitatory components $w_{E}^{\text{loc}}+w_{E}^{\text{lat}}$

$$\begin{array}{c} B_{E}=1/[\frac{1}{2\pi RW_{\text{ex}}^{2}}+H\left(0,\Lambda ,RW_{\text{ex}}\right)+H\left(0,2\Lambda ,RW_{\text{ex}}\right)]. \end{array}$$ (14)

We now define the normalized combined excitatory profile as

$$\begin{array}{c} w_{E}=B_{E}\left(w_{E}^{\text{loc}}+w_{E}^{\text{lat}}\right), \end{array}$$ (15)

which by design has zero-order Fourier mode of 1. The complete connectivity function is

$$\begin{array}{c} w\left(r\right)=P\;[w_{E}\left(r\right)+\left(C-1\right)w_{I}\left(r\right)], \end{array}$$ (16)

where *C* is a constant controlling the relative strength of excitation and inhibition. When *C* = 0 excitation and inhibition are balanced and when *C* is negative there is net inhibition globally. We introduce a constant *P* that matches the value of *W*’s largest Fourier mode with the connectivity used in [41]. This final scaling of the overall connectivity allows us to manipulate any of the connectivity parameters, whilst keeping all non-connectivity parameters constant (e.g. input and threshold function parameters). Failing to do this means that the correct operating region of the model would shift each time, say, *RW*_ex_ was modified. Two constants *g*_ex_ and *g*_in_ in (3)–(4) are given by

$$\begin{array}{c} g_{\text{ex}}=B_{E}P,\;\text{and}\;g_{\text{in}}=P\left(C-1\right). \end{array}$$ (17)

### Orientation preference map

Retinotopic space is mapped to the surface of V1 [47] and different locations show preference for a variety of low-level visual features such as spatial frequency, ocular dominance, orientation and direction of motion [48, 49]. Cortical neurons exhibiting similar preference for low-level features are organized in a columnar fashion [50, 51]. Orientation preference varies incrementally along the cortical surface [52], defining a quasi-periodic organization characterized by linear zones and pinwheels, singularities about which all orientation preferences are present along a circular path [18]. The orientation maps have a quasi-periodic organisation with a regular length scale of around 0.5–1 mm (depending on species and cortical areas), which can be measured as the mean distance between iso-orientation domains. This length scale, which we denote *Λ*, is reflected as a peak in the Fourier spectrum of the preference map represented as polar argument [53, 46]. Orientation preference can be deduced from single-electrode recordings [54] or with optical imaging of intrinsic signals [18, 10].

The characteristics, artificial generation and biological development of orientation preference maps have been investigated in various modelling studies [55, 56, 46, 57]. [56] showed the importance of long range connections in generating the quasi-periodic repetition of key map features in a canonical pattern forming system with spatially extended complex representation of orientation. [46] showed that when normalized by the regular length scale, orientation preference maps show a constant pinwheel density; see also [58, 59]. [57] explored mechanisms for the stable development using a Hebbian learning method for connections in a two-stage (LGN, V1) model and found an important role for adaptation and normalisation.

In this study we generated realistic maps specifically for our planar model with discrete representation of orientation (in four-sub-populations). The maps were generated specifically for the periodic domain used in our model using a spatial Hebbian-like learning rule operating on the converged model output $u_{i,n}^{\text{fin}}$ from a series of localized inputs *I*_*i*_ with random orientations and at random locations. After each simulation the *J*_*i*_ were updated via the following rule:

$$\begin{array}{l} J_{i,n+1}=J_{i,n}+H_{a}I_{i}\;(1-{\langle \left|J_{i,n}\right|⋆G\rangle }_{\left[0,1\right]})u_{i,n}^{\text{fin}},\\ J_{i,n+1}={\langle J_{i,n+1}\rangle }_{\left[-1,1\right]}. \end{array}$$

*H*_*a*_ is the learning rate, *G* a smoothing kernel (8) with *σ* = 0.6*Λ*, and 〈.〉 rectification on the given interval. The smoothing ensures that regions that already have local structure are modified less than regions without local structure. Learning was initiated from a homogenous initial set of *J*_*i*_ = 0 (where the model produces localized multi-bump states in the stimulated area and sub-population). The learning process converged (e.g. pinwheel density stabilizes) after around 1600 steps as the map takes on structure across the whole domain (after this the term 1 − 〈|*J*_*i*,*n*_| ⋆ *G*〉_[0, 1]_ remains close to 0 across the whole domain, although small changes in the map continue). Final maps (at 6400 steps) were high-pass and low-pass filtered as described in [46]. The maps used in this study are included as part of the simulation code (see Supplemental information S1 Code), thus allowing the results to be independently reproduced. Full details of the method will be the subject of a separate study.

**Fig 2:**
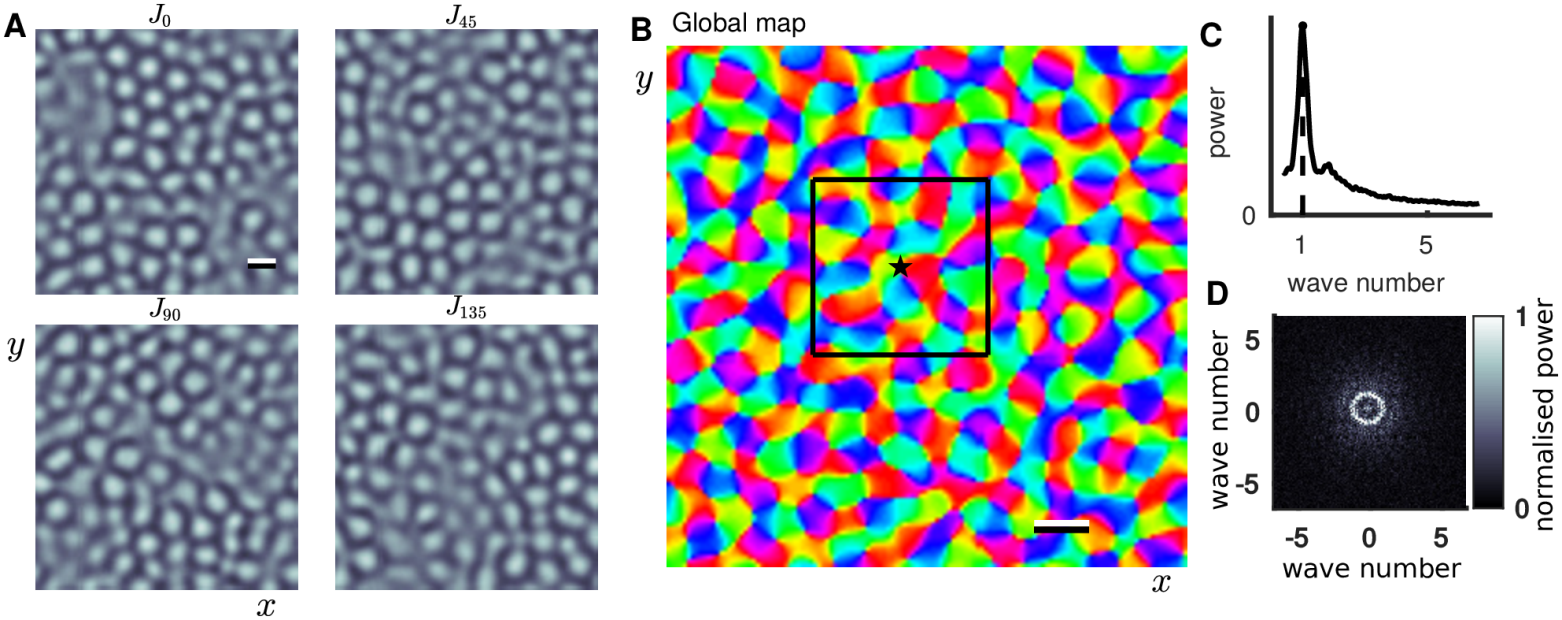
Orientation preference map. **A:** Four component maps, each associated with a different orientation. Lighter (darker) regions correspond to higher (lower) selectivity for the specific orientation. Scale bar is *Λ*. **B:** Composite preference map, colormap shows orientation preference. Star and black box show the map location studied in more detail in Fig 3. Model simulations in the results section were computed at different locations randomly chosen from this global map. Scale bar is *Λ*. **C:** Spectral power as a function of radial wave number for the map in B (computed as in equation 7 of [46] supplementary materials) showing a sharp peak corresponding to the regular length scale *Λ*. **D:** 2D power spectrum; normalised by peak value in C.

Alternatively, one could use maps obtained experimentally directly with our model, but this would not have any significant effect on the results presented. For the purposes of the present study it is sufficient to show that the maps used in our model show characteristic features of realistic maps including linear zones, pinwheels and the regular length scale *Λ*. Fig 2A shows the component maps *J*_*i*_, each associated with a different orientation, that in (1)–(4) modulate inputs with strength *β*_inp_ and long range excitatory connections with strength *β*_rec_. In conjunction, the composite maps combine to give the orientation preference map shown in Fig 2B, which has a regular length scale *Λ* characterized by a sharp peak in the map’s spectral power curve (Fig 2B).

### Anatomically motivated parameter operating range for the model

The effective tuning of lateral connections is computed across ranges of the connectivity parameters *RW*_ex_ (width of peaks in excitation) and *β*_rec_ (orientation bias of long-range lateral connections). These parameters were chosen as they have a strong effect on the anatomical measure of interest over ranges where the model is well defined. Another choice could be *ζ* (controlling the decay of peaks in excitation), but this would be redundant with *ζ* having a similar effect as varying *RW*_ex_. These computations allow for a direct comparison with the anatomical-data-based model analysis presented in [14], which reported the orientation bias of long-range lateral connections in V1 L2/3. Optical imaging was used to find orientation preference maps and combined with intracellular labeling of lateral projections of pyramidal cells to identify target locations relative to the preference map. The orientation bias was quantified by tuning curves of the orientation preference at axon terminals relative to orientation preference in a region local to the originating cell’s body. Tuning curves were quantified by parameter-fits to a von-Mises distribution (circular normal distribution). Our model was developed to be able to provide a direct point of quantitative comparison with these measures. The distribution is defined on a circular domain and parametrized by a tuning coefficient *κ* ≥ 0 (0 is untuned, larger is more tightly tuned) and a preferred orientation *μ* ∈ [0, 180):

$$\begin{array}{c} f\left(x;\mu ,\kappa \right)=\frac{e^{\kappa \cos(x-\mu )}}{2\pi I_{0}\left(\kappa \right)}. \end{array}$$ (18)

*I*_0_(*κ*) is the modified Bessel function of order 0. [14] reported values of *κ* in the range 0.7–1.2 for population-level tracing of lateral connections.

**Fig 3:**
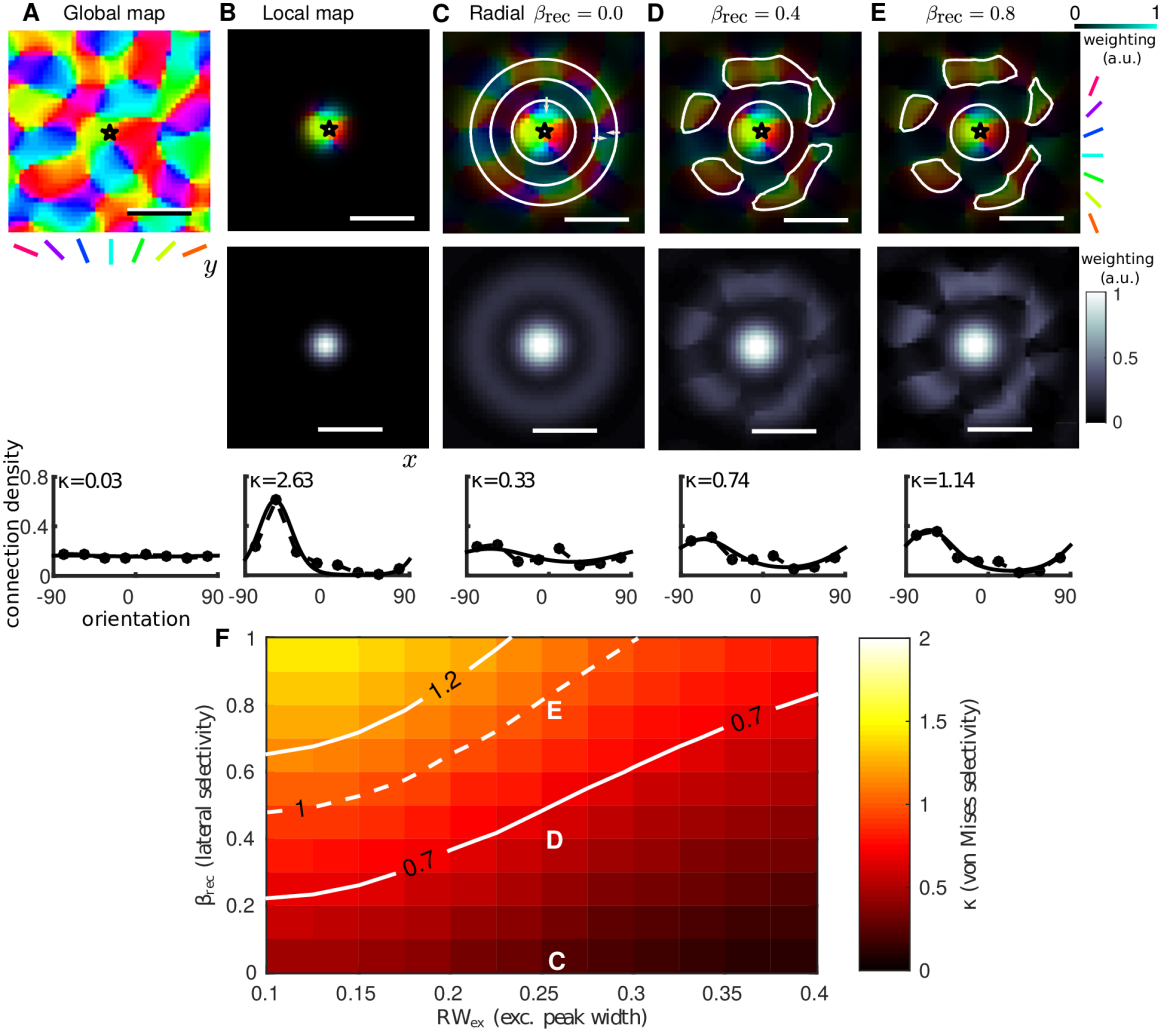
Effective orientation tuning of lateral connections dependent on model parameters. **A:** (top) Portion of the global orientation map (black box in Fig 2B). (bottom) Number of pixels (normalized) selective for each orientation for eight equally spaced bins in [−90, 90]. The tuning curve (dashed with markers) for the global map and best-fit von-Mises distribution (solid) shows no orientation bias (*κ* ≈ 0). **B:** (top) Local region weighted by a Gaussian function (middle) centered at *, color intensity reflects the weighting. (bottom) Scaling the pixel counts by the weighting function reveals the strong local orientation tuning. **C–E:** (top) Effective lateral connections from * for *RW*_ex_ = 0.25 and different values of *β*_rec_. White contours highlight regions with most connections. In C White arrows indicate central circle and annular region where there are more connections (less connections in the darker region between). (middle) Excitatory connectivity profile used as weighting. The profile of lateral connections is purely radial when *β*_rec_ = 0, and, when *β*_rec_ > 0, biased toward regions with orientation preference matching that of the local map (B). (bottom) Corresponding orientation tuning curves for lateral connections. Tuning strength *κ* increases with *β*_rec_. **F:** Colormap shows the mean value of *κ* (averaged from 50 map locations, one example shown in A–E) across range of *β*_rec_- and *RW*_ex_-values. Parameter values for panels C, D and E are labeled. Solid white contours (*κ* = 0.7, *κ* = 1.2) correspond to range of values reported in [14].

We perform a similar computation for the connectivity function in our model. The computation of the effective orientation tuning of lateral connections, at one specific map location (Fig 3A(top)), is illustrated in Fig 3A–3E. For the orientation preference map one can compute an orientation tuning curve by counting the number of pixels falling within equally sized orientation bins, as shown in Fig 3A (bottom, circular markers). All orientations are represented with equal probability so the profile is untuned and a best-fit von-Mises distribution (solid curve) has *κ* ≈ 0 (fit determined using a least-squares minimisation with Matlab’s lsqcurvefit for *κ* and *μ* in (18)). A spatial weighting for the pixel count can be introduced in order to find the tuning of orientations in some local region. A 2D Gaussian function (Fig 3B(middle)) was used to give the local weighting shown in Fig 3B(top). In most regions of the map this results in a sharp tuning (note large *κ*) centered at one specific orientation as shown in Fig 3B(bottom). Introducing the radial profile of excitatory connections from the model as a weighting function (Fig 3C(middle), see (15)) one can compute the effective tuning of the lateral connections. With *β*_rec_ = 0 the weighting function is radially symmetric, nevertheless there is an expected weak tuning of the weighted connections (Fig 3C(bottom)) due to the structure of the orientation preference map; there is a slight bias toward finding similar orientations at a range *Λ* (annular ring) relative to the origin (star). Increasing *β*_rec_ introduces an orientation-specific bias in the weighting profile for the long-range connections (Fig 3D–3E(middle)). This results in locations with similar orientations to the origin being targeted specifically by long-range connections (Fig 3D–3E(top)) and a corresponding increase in the tuning strength *κ* (Fig 3D–3E(bottom)).

As one might expect the tuning strength of connections (*κ*) increases monotonically with increasing *β*_rec_ or decreasing *RW*_ex_, which allows us to define an operating range for the model before running simulations. Fig 3F shows *κ* (average value from 50 randomly selected map locations) computed at combinations of *RW*_ex_ ∈ [0.1, 0.4] and *β*_rec_ ∈ [0, 1]. Map locations close to pinwheels (7/50) identified by having a local tuning (computed as in Fig 3B) with *κ* < 1 were excluded (this exclusion had a very minor effect). The map shows that *κ* increases with *β*_rec_ (as expected) and decreases with *RW*_ex_. Solid white contours show a band of values for *RW*_ex_ and *β*_rec_ where the tuning of connections in the model is consistent with the anatomical data (*κ* ∈ [0.7, 1.2]). These contours are later replotted in *Orientation selective activation is restricted to stimulus footprint in the anatomical parameter range* and *Reduced inhibition leads to orientation selective activation outside stimulus footprint* for comparison with model simulations. For *RW*_ex_ much beyond 0.4 the anatomical data cannot be matched as *β*_rec_ must be <1.

### Conversion of model output to VSD-like signal

**Fig 4:**
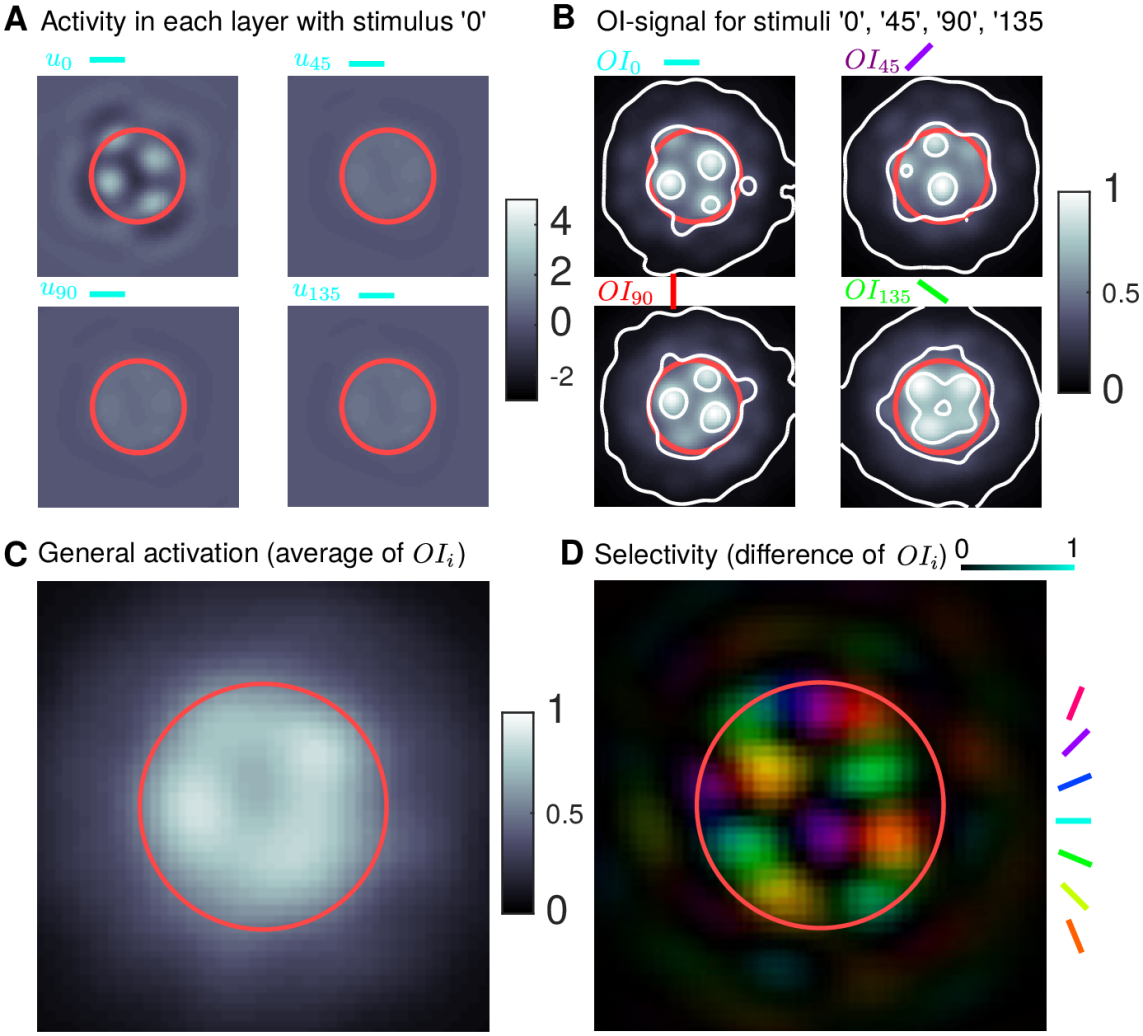
Conversion of model output to VSD-like signal. **A:** Example converged model response for a radial input (FFF in red) with orientation 0° (simulated for 600 ms). The corresponding sub-population *u*_0_ responds with a multi-bump pattern with the location of the spots determined by *J*_0_, whilst the other sub-populations have a smaller response. **B:** VSD-like signal *OI* for stimuli with different orientations. For the orientation 0° the signal *OI*_0_ (top left) is computed via (19) from the four sub-population responses as shown in A. Each panel is generated from a single model simulation, like the one in A, but with a different orientation. Contours at *OI_i_* = {0.07, 0.45, 0.8} show roughly the broad long-range activation, the plateau close to the stimulus FFF and localized bumps of activity on the plateau. **C:** The general activation, computed via (21) as the average of the *OI*-responses in B. **D:** Selectivity (color intensity, dark is non-selective) and preferred orientation (color) computed via (25) and (24) by taking as difference maps from the *OI*-responses in B.

The process of converting the model output from individual simulations into a VSD-like signal, and the method for computing the general and orientation selective activation, is illustrated in Fig 4. For a given oriented simulation, the *I*_*i*_ are weighted toward the specific orientation (Fig 1A). The input is further modulated by *J*_*i*_ if *β*_inp_ > 0 (Fig 1B) and recurrent connections in each sub-population are modulated by *J*_*i*_ if *β*_rec_ > 0. The sub-population corresponding to the input orientation responds above threshold in a multi-bump pattern with the location of the bumps determined by *J*_*i*_, whilst the other sub-populations have a sub-threshold response (Fig 4A). We first transform the sub-population variables *u*_*i*_ into a VSD-like signal following a similar method to the one proposed in [38]. The sub-population membrane potentials in Fig 4A are converted into a firing rate, processed through weighted excitatory and inhibitory connectivity profiles, summed across the sub-populations and diffused with a Gaussian profile (representing the attenuation and diffusion of the signal in cortical tissue). The contribution from inhibition is assumed to be in the range 15–20% [60, 61]. The resulting optical imaging signal *OI*_0_ in Fig 4B(top left) was computed from the four sub-population responses in Fig 4A. For different orientation inputs, the other *OI* signals can be computed in a similar fashion (Fig 4B, other panels). The general activation Act is computed as an average across the responses to inputs with four different orientations; the response in Fig 4C was computed as an average of the four responses in Fig 4B. The preference Pref and selectivity Sel (Fig 4D) are computed by transforming the four *OI* signals into polar coordinates from difference maps (*OI*_0_ − *OI*_90_ and *OI*_45_ − *OI*_135_) where the angular coordinate (argument) is the preference and the radial coordinate (magnitude) is the selectivity [62]. Thresholds delineating the general activated area and orientation selective area are defined as a fraction of the average activation or selectivity inside the FFF. The radial profiles (average radial decay) of Act and Sel are characterized by the parameters of best-fit Naka-Rushton functions [63] (a widely-used, smooth, monotonically decaying function (29), which has been used to fit, e.g., contrast response data [64]). Further details and equations of all the processing steps are given below.

Following the method described in [38], to account for the known optical diffusion of the VSD signal (light) in V1 L2/3, we convolved the signal with a Gaussian distribution (8) with *σ*_OI_ = 0.075*Λ*. Note that the results in this study are not contingent on this specific value, there being little effect of either increasing or decreasing *σ*_OI_ by a factor of 2. The unattenuated imaging signal *u*_*i*_ for each sub-population is assumed proportional to the post-synaptic membrane potential. Hence we first computed the dynamics of mean pre-synaptic membrane potential for each sub-population *u*_*i*_ as given by (1)–(4), which is converted to a firing rate of the pre-synaptic neurons through *S*. The postsynatpic population response was then computed via a convolution of the presynaptic firing rate with the connection profile. There is an 85% contribution from excitation and a 15% contribution from inhibition, leading to a weighting for inhibition of *p*_*I*_ = 0.177. These are summed across the sub-populations *i* to give the total unattenuated signal (expression in large parentheses). Finally the optical imaging (OI) signal is computed as a convolution of the total unattenuated signal with a Gaussian:

$$\begin{array}{c} OI\left(x,y\right)=(\sum_{i}\;S\left(u_{i}\left(x,y\right)\right)\overset{\left(x,y\right)}{⋆}[w_{E}^{\text{loc}}\left(x,y\right)-p_{I}w_{I}\left(x,y\right)+w_{E}^{\text{lat}}\left(x,y\right)\left(1+\beta_{\text{rec}}J_{i}\right)])\overset{\left(x,y\right)}{⋆}g\left(x,y,\sigma_{\text{OI}}\right). \end{array}$$ (19)

This equation converts the model’s state variables *u*_*i*_ for an input stimulus with specific orientation (e.g. 0° as in Fig 4A) into a VSD-like signal (e.g. *OI*_0_ in Fig 4B, top left).

We are interested to explore how cortical activation spreads over time. One potential issue with the temporal dynamics in the model, and (19), is the assumption that activity generated through long range lateral connections ($w_{E}^{\text{lat}}$) propagate instantaneously. Although the model converges to the correct final state, the transient dynamics may not be captured exactly. To solve this, whilst avoiding the introduction of say delay terms in (1)–(4), we assume that there is a slower timescale *τ*_lat_ = 240 ms for the portion of the *OI*-signal generated through $w_{E}^{\text{lat}}$:

$$\begin{array}{c} OI\left(x,y,t\right)=(\sum_{i}\;S\left(u_{i}\left(x,y,t\right)\right)\overset{\left(x,y\right)}{⋆}[w_{E}^{\text{loc}}\left(x,y\right)-p_{I}w_{I}\left(x,y\right)+\left(1-e^{-t/\tau_{\text{lat}}}\right)w_{E}^{\text{lat}}\left(x,y\right)\left(1+\beta_{\text{rec}}J_{i}\right)])\overset{\left(x,y\right)}{⋆}g\left(x,y,\sigma_{\text{OI}}\right), \end{array}$$ (20)

where *t* is the time after stimulus onset. The introduction of *τ*_lat_ is done at the post-processing stage only and its value was chosen to match data from [24].

For four sequential simulations, each with an input with different orientation, the *OI* signal can be computed (Fig 4B). The general activation is the average of these signals:

$$\begin{array}{c} \text{Act}\left(x,y,t\right)=\frac{1}{4}\;\sum_{j}OI_{j}\left(x,y,t\right),\;j=0,45,90,135, \end{array}$$ (21)

as shown in Fig 4C.

Before computing the preference Pref and selectivity Sel, the VSD signals are normalized by a scale factor that accounts for differences in the maximum value over (*x*, *y*) across the four simulations with different orientations. Two difference maps between the normalized VSD signals from simulations with orthogonal inputs are computed:

$$D_{1}\left(x,y,t\right)=OI_{0}\left(x,y,t\right)-OI_{90}\left(x,y,t\right),$$ (22)

$$D_{2}\left(x,y,t\right)=OI_{45}\left(x,y,t\right)-OI_{135}\left(x,y,t\right).$$ (23)

The orientation preference of the activation is given by

$$\begin{array}{c} \text{Pref}\left(x,y,t\right)=\text{Arctan}(D_{1}\left(x,y,t\right),D_{2}\left(x,y,t\right)), \end{array}$$ (24)

where Arctan is the four quadrant inverse tangent, and the selectivity strength is given by

$$\begin{array}{c} \text{Sel}\left(x,y,t\right)=\sqrt{D_{1}{\left(x,y,t\right)}^{2}+D_{2}{\left(x,y,t\right)}^{2}}. \end{array}$$ (25)

In the results section, all plots of Sel(*x*, *y*, *t*) and Act(*x*, *y*, *t*) are scaled by 1.1× their values at the final time point *t*_final_ in the simulation, thus showing the time-evolution relative to the final state. The thresholds contours *T*_*act*_ and *T*_*sel*_ for activation and selectivity (and corresponding areas *A*_*act*_ and *A*_*sel*_) are set as a fraction of the mean activation and selectivity within the FFF at the final time point,

$${\bar{\text{Act}}}_{\text{FF}}=\underset{r<r_{\text{FF}}}{\text{mean}}\left(\text{Act}\left(x,y,t_{\text{final}}\right)\right),$$ (26)

$${\bar{\text{Sel}}}_{\text{FF}}=\underset{r<r_{\text{FF}}}{\text{mean}}\left(\text{Sel}\left(x,y,t_{\text{final}}\right)\right),$$ (27)

where *r*_FF_ is the FFF boundary. The thresholds are given by

$$\begin{array}{c} T_{act}=\eta_{\text{Act}}{\bar{\text{Act}}}_{\text{FF}},\\ T_{sel}=\eta_{\text{Sel}}{\bar{\text{Sel}}}_{\text{FF}}, \end{array}$$ (28)

where *η*_Act_ = 0.2 and *η*_Sel_ = 0.5. These thresholds were chosen ad hoc. In order to ensure the results obtained weren’t contingent only on these choices, we further characterized the radial decay rates of the general and orientation selective activation.

We also look at radial profiles of the general and selective activation patterns (radial decay of Act and Sel). Radial profiles Act(*r*, *t*) and Sel(*r*, *t*) are computed by re-meshing Act(*x*, *y*, *t*) and Sel(*x*, *y*, *t*) on radial coordinate system (*r*, *θ*) centered at the stimulus center and averaging in the angular coordinate *θ*. The decreasing Naka-Rushton function *NR* used here decays to zero as *r* increases:

$$\begin{array}{c} NR\left(r\right)=R_{\text{max}}\;(1-\frac{r^{n}}{r^{n}+r_{50}^{n}}), \end{array}$$ (29)

where the exponent *n* > 0 describes the steepness, the maximal value is *R*_max_ and the half-max *r*-value is *r*_50_ > 0. Best-fit Naka-Rushton functions were determined by minimising, for example the least-squares distance between Act(*r*, *t*_final_) and *NR*(*r*) varying the parameters *n*, *R*_max_ and *r*_50_ using Matlab’s lsqcurvefit function.

## Results

### Radial profile of lateral connections determines the extent of orientation selective activation

The spatio-temporal dynamics are investigated for a specific case with inputs modulated by the orientation preference map (*β*_inp_ = 0.25) but with radially symmetric lateral connections (*β*_rec_ = 0, no orientation bias in lateral connections). The dynamics produced by the model will be illustrated for two values of the width of the peaks of excitatory connections *RW*_ex_. When *RW*_ex_ is small, excitatory connections cluster on tightly on rings at distances *Λ* and 2*Λ* away from the origin. When *RW*_ex_ is larger the clustering at these specific ranges is more diffuse (Fig 1G).

**Fig 5:**
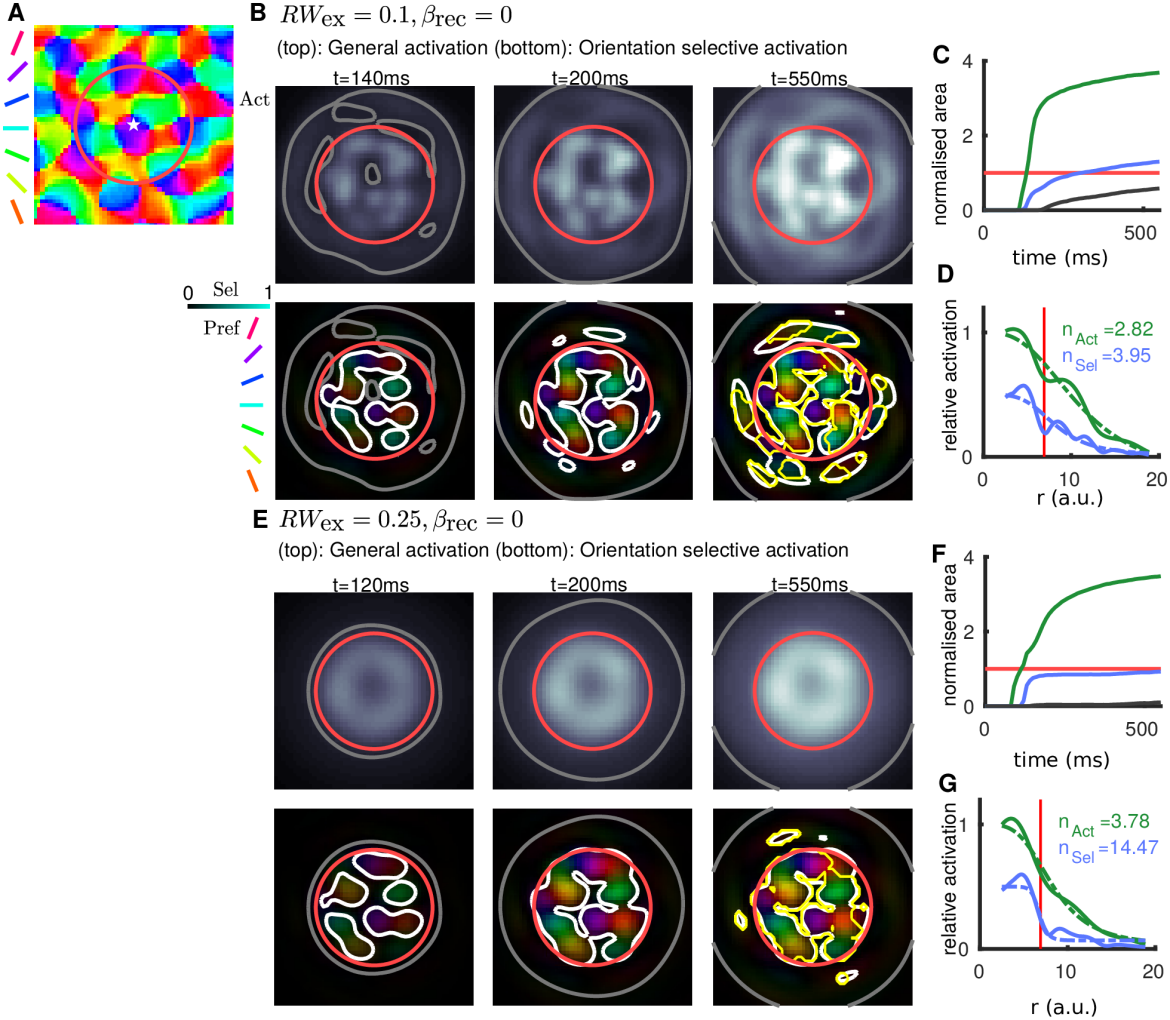
Temporal dynamics and effect of varying *RW*_ex_. **A:** Stimulus location (star) and (FFF) (red circle) relative to the orientation preference map. Note the orientation preference map modulates the inputs (*β*_inp_ = 0.25) but not the recurrent lateral excitatory connections (*β*_rec_ = 0) in these simulations. **B–D:** *RW*_ex_ = 0.1. **E–G:** *RW*_ex_ = 0.25. **B,E:** (top) Greyscale map shows time snapshots of the general (averaged) activation, grey contour marks the limit of the activation, which expands with time, red circle is the FFF. (bottom) Colormaps show time snapshots of orientation selective activation, the color indicates preferred orientation and color intensity indicates the degree of selectivity (dark regions are not selective). Grey contour replotted from top panels; white contours highlight the component of this activation that is significantly selective for orientation. Yellow contour in bottom right panel only highlights the component significantly selective for orientation *and* with preference matching (±30°) the underlying map in A. **C,F:** Temporal dynamics of the activated area broken down into general (green, area inside grey contour in B,E), selective (blue, area inside white contour in B,E) and selective outside the FFF (black, area of white contour outside red circle in B,E). Red line is the FFF area. **D,G:** Radial profile of the general activation (solid green) and orientation selective activation (solid blue) averaged in angular coordinate about the center of the stimulated region. Best-fit Naka-Rushton functions are dashed with exponent *n* as indicated. Vertical red line is the FFF radius. **Multimedia:** Animations of the simulations shown in panels B and E are available in Supplemental information S1 Video.

Simulations were run, sequentially for four different orientations, with a radial input centered at a specific location in the orientation preference map (Fig 5A). Fig 5B and 5E (top) show that the general (averaged) activation spreads outward from the center of the stimulated region (note that simulations are computed on a domain around 3 times larger than the window shown here). Only some portion of this general spread of activation is orientation selective as shown in the bottom panels (area inside white contour is significantly smaller than area inside grey contour). When, *RW*_ex_ is small the selective activation extends outside the FFF (Fig 5B(bottom,right)), and when *RW*_ex_ is larger it is confined to the FFF (Fig 5E(bottom,right)). The temporal dynamics of the general and orientation selective areas (Fig 5C and 5F) shows that the rate of the area increase slows down after roughly the first 100 ms. Nevertheless, when *RW*_ex_ is small the spread of activation persists after 550 ms as shown by the blue/green curves still increasing in Fig 5C (eventually converges at ∼ 700 ms). When *RW*_ex_ is larger the orientation selective activation converges after around 200 ms although the general activation continues to increase slowly (Fig 5F). This latter behavior is compatible with the observations made using VSDI in [24]. The radial profile of the general and selective activation shows the decay with distance from the center of the stimulated region (Fig 5D and 5G). When *RW*_ex_ is small, the profile of the general and selective activation exhibits a similar decay profile as marked by similar values of the exponent *n* in a best-fit Naka-Rushton function for the two profiles (Fig 5D). For a larger *RW*_ex_ the exponent for the orientation selective activation is substantially larger (*n*_Sel_ = 14.47), indicating a steep transition from high to low selectivity, than for the general activation (Fig 5G).

Even with lateral connections not modulated by the orientation preference map (no orientation bias in lateral connections), orientation selective activation can be generated outside the FFF of the stimulus due to convergent excitation generated from regions directly stimulated within the FFF. Indeed, activated locations with the same preference mutually excite each other at a range *Λ*. These activated regions can further generate overlapping excitation at *Λ*-equidistant points, either inside the FFF, or potentially outside the FFF. Orientation selective activation generated in this way, outside the FFF, does not necessarily agree with the orientation preference map (Fig 5B, bottom right, region inside the white contour but not the yellow). This occurs if the range of peaks in excitation are highly specific (small *RW*_ex_) as in Fig 5B–5D. These findings illustrate that with *RW*_ex_ excessively small, the ringed connectivity can lead to undesired behaviour that is inconsistent with activation observed in experiments. We note that later in section *Reduced inhibition leads to orientation selective activation outside stimulus footprint* we find that spurious activation (not agreeing with the preference map) can be generated by another mechanism (destabilization of activity). When the peaks in excitation are broader (*RW*_ex_ > 0.2), the orientation selective activation decays quickly at the border of the stimulated region and no such miss-tuned activation (as generated by convergent excitation) is observed outside the FFF.

We have seen how our modelling results allow us to distinguish between patterns of activation that are consistent with imaging studies in terms of 1) the area and range of general (grey) and selective (white) activation relative to the FFF (red) in Fig 5B and 5E(bottom); 2) whether activation reflects the correct orientation with respect to the underlying preference map (difference between yellow and white contours in Fig 5B and 5E(bottom,right); and 3) the rate of decay of general and selective activation in Fig 5D and 5G as quantified by *n* (larger is a steeper decay). The simulation shown in Fig 5E–5G is consistent with [23] and [24] because 1) in E(bottom,right) the grey contour extends much further than the white, 2) in E(bottom,right) the yellow and white contours agree closely, 3) in G the exponent *n* is larger for the selective activation (blue) than for the general activation (green). Further, this steep decay of selectivity is consistent with there being a plateau of highly selective activation inside the FFF that transitions sharply to low selectivity outside the FFF.

### Orientation bias of lateral connections increases the range of orientation selective activation

**Fig 6:**
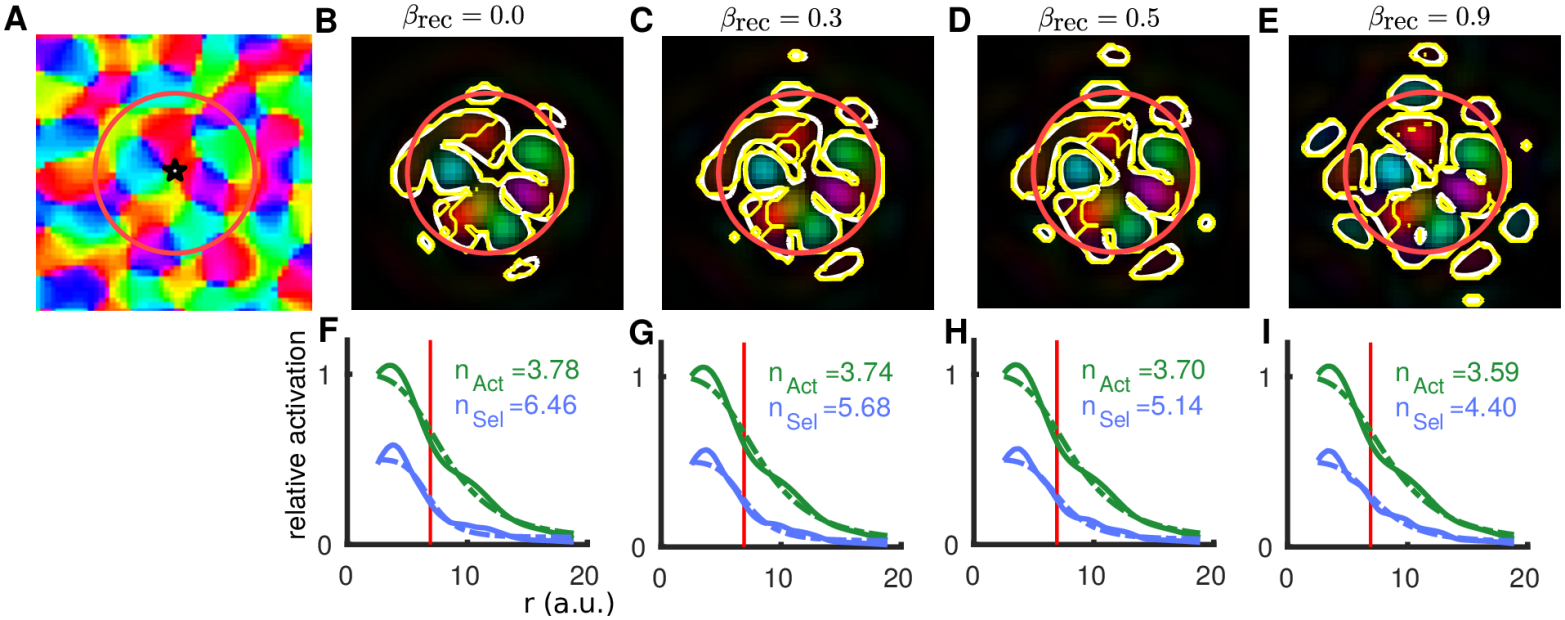
Spatial spread of orientation selective activation varying *β*_rec_ (fixed *RW*_ex_ = 0.25). **A:** Stimulus location (star) and FFF (red circle) relative to the orientation preference map. The map modulates the inputs (*β*_inp_ = 0.25) and the lateral excitatory connections dependent on *β*_rec_. **B–E:** Colormaps show the converged (*t* = 550 ms) profile of orientation selective activation (as Fig 5B and 5E (bottom row)) for indicated values of *β*_rec_. White contours highlight the component of this activation that is significantly selective for orientation. Yellow contour in bottom right panel only highlights the component significantly selective for orientation *and* with preference matching (±30°) the underlying map in A. **F–I:** Radial profile of the general activation (solid green) and orientation selective activation (solid blue), best-fit Naka-Rushton functions with exponents *n* are dashed (as Fig 5D and 5G).

The effect of introducing an orientation bias to the long-range lateral connections (*β*_rec_ > 0) is shown in Fig 6. For another location in the orientation preference map (Fig 6A) the orientation selective activation is shown for different values of *β*_rec_ in Fig 6B–6D with the radial profile shown in F–I. The parameter values for panels Fig 6B and 6F are the same as those for Fig 5E–5G (*RW*_ex_ = 0.25 and *β*_rec_ = 0), the only difference is the location in the orientation preference map. These two examples show a qualitatively similar spatial profile, and in general, the resulting spatio-temporal dynamics are independent of the location in the orientation preference map (five locations were randomly chosen for the results in this paper). Across the five locations there is a large range of *n*_Sel_ (between 6 and 15) when *RW*_ex_ = 0.25 and *β*_rec_ = 0. However, this variability in *n*_Sel_ across locations is much less (between 5 and 7) when *β*_rec_ is increased to say 0.5. As *β*_rec_ is increased, more orientation selective activation is observed increasing from small isolated patches outside the FFF at intermediate values (Fig 6C–6D) to many larger for *β*_rec_ = 0.9 (Fig 6E). In contrast with the orientation selective activation outside the FFF observed in Fig 5B(bottom), which does not necessarily reflect the local orientation from the preference map, the activation outside the FFF in Fig 6E reflects the preference map (white and yellow contours agree) as it is generated by orientation-biased long-range connections. The increased selective activation outside of the FFF coincides with a reduced slope (smaller exponent *n*) in the radial decay of selective activation shown in Fig 6F–6I.

Again, the results shown here for *β*_rec_ in the range [0, 0.5] are consistent with imaging studies [23, 24], as the region of selective activation is predominantly confined to the FFF (limited small selective regions outside the red circle), the preference agrees with the underlying map (yellow and white contours are very similar) and the exponent *n* is larger for the selective activation.

### Orientation selective activation is restricted to stimulus footprint in the anatomical parameter range

**Fig 7:**
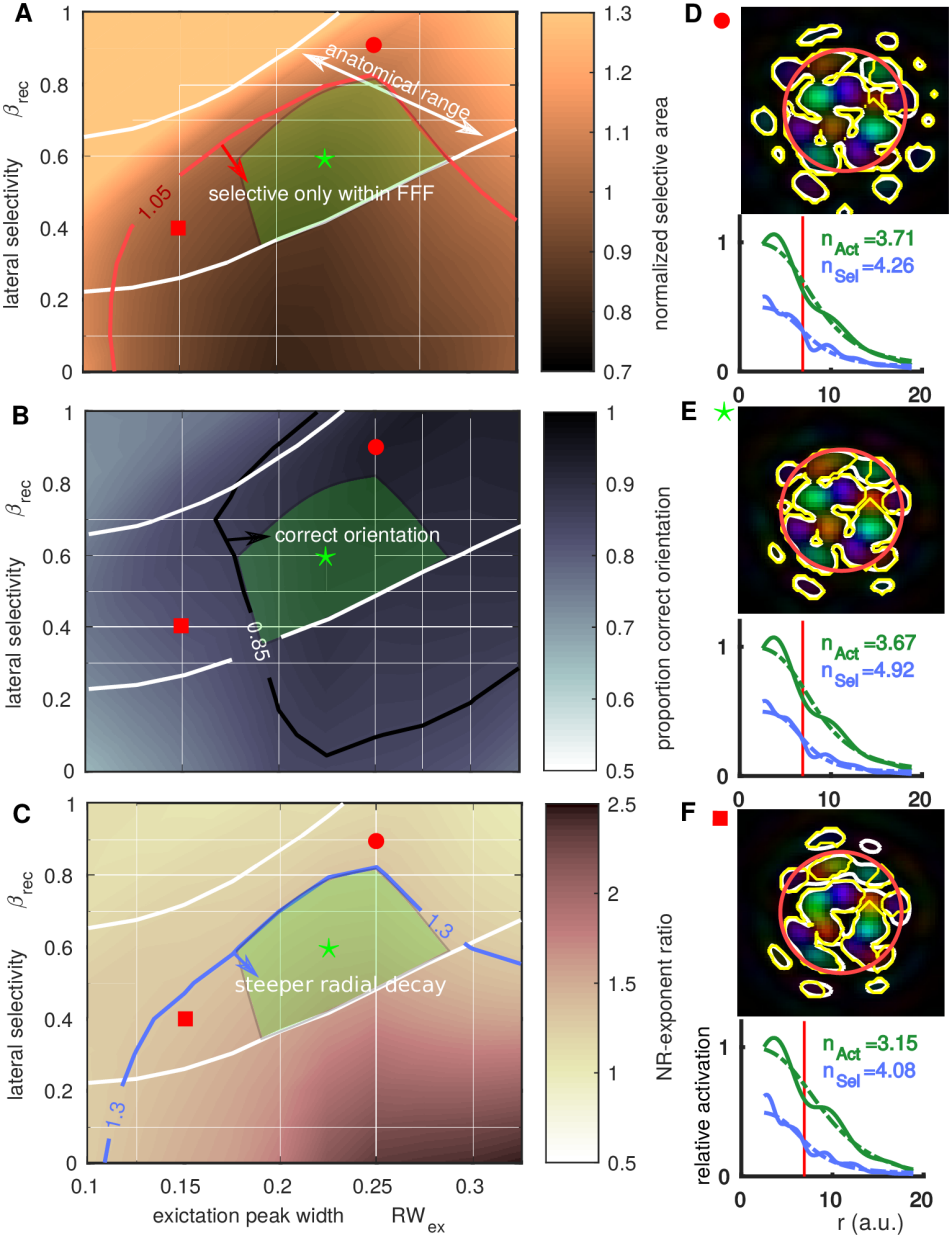
Profile of orientation selective activation relative to anatomical parameter range. In A–C the green triangular region (defined in C) is the proposed operating regime for the model, in agreement with both anatomy and imaging studies. **A:** Colormap shows the normalized selective area (relative to the FFF) across relevant ranges of *RW*_ex_ and *β*_rec_ (averaged across 5 simulations at different preference map locations). White contours indicate the anatomically relevant operating range for the model as determined in Fig 3 (in agreement with available anatomical data). Within the red counter (green arrow) the orientation selective activation is confined to the FFF of the stimulus. **B:** Colormap of the proportion of selective area with the correct orientation preference (normalized difference between white and yellow contours in D-F). In the top-right quadrant (inside contour with green arrow), the majority of the activation reflects the correct orientation. **C:** Map shows how rapidly orientation selective activation decays moving out from the center of the stimulated region (dark region is steeper). Quantified by the ratio *n*_Sel_/*n*_Act_ of the exponents of best-fit Naka-Rushton functions for the radial profile of the selective and general activation, see D–F. Other individual examples were shown in Figs 5D and 5G and 6F–6I. In the green triangle, there is steep decay, selectivity within the FFF (A), the correct orientation (B), and parameters are within the anatomical range. **D:** Example of final snapshot of the orientation selective activation (*t* = 550 ms) and radial profile (bottom) for *RW*_ex_ = 0.25 with *β* = 0.9 (green circle in A–C), where there is significant activation outside the FFF. **E:** As D with *RW*_ex_ = 0.225, *β* = 0.6 (green star in A–C), where the activation is confined to the FFF. **F:** As D with *RW*_ex_ = 0.15, *β* = 0.4 (red square in A–C); note spurious activation at 1 o’clock. **Multimedia:** Animations of the simulations shown in panels D, E and F are available in Supplemental information S1 Video.

**Fig 8:**
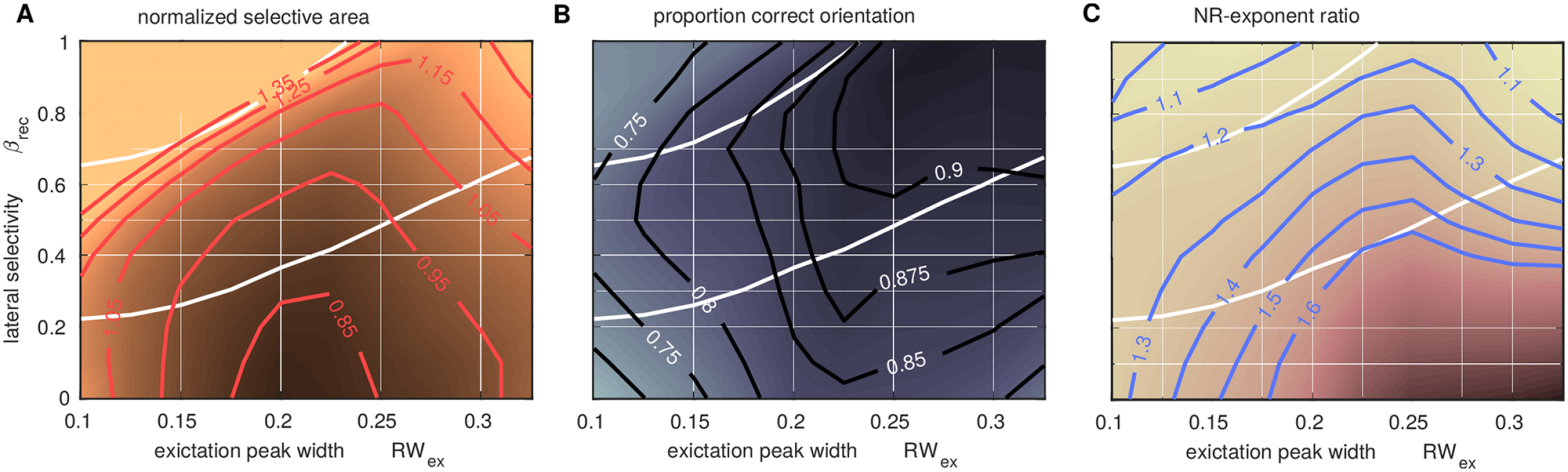
Dependence of operating region on thresholds. Colormaps as in Fig 7A–7C with additional contours. **A:** Decreasing the threshold for normalized selective area to 0.95 would still give a reasonable operating region. **B:** Similarly for a marginal increase, to say 87.5%, in the proportion of correction orientation threshold. **C:** Similarly for an increase in the exponent ratio to, say, *n*_Sel_/*n*_Act_ = 1.45.

**Fig 9:**
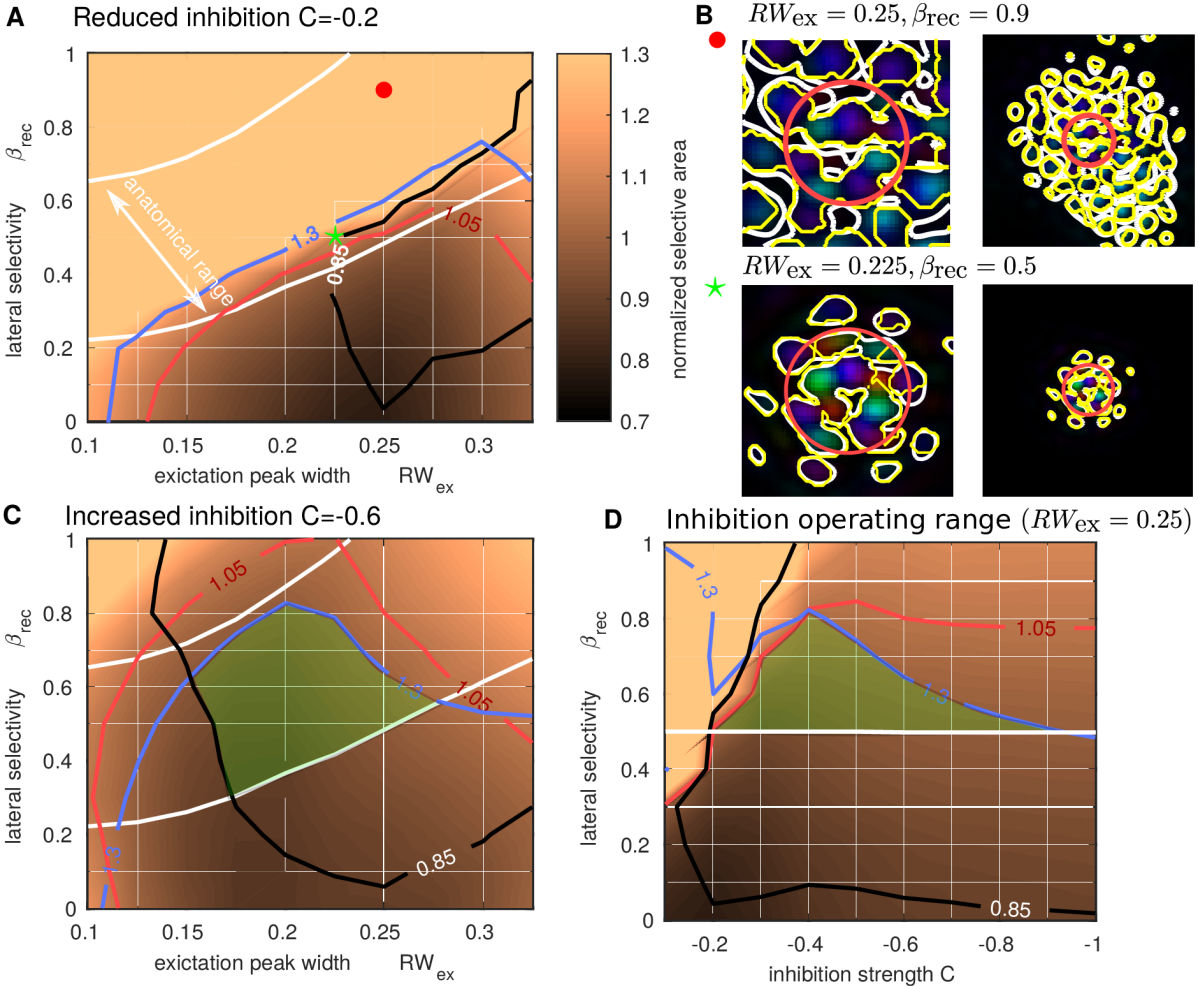
Reduced inhibition case: Anatomical parameter range and profile of orientation selective activation. **A:** As Fig 7A with the strength of inhibition reduced by 50%. There is no overlap between the anatomical range and the region where activation is confined to the FFF of the stimulus (inside the red contour). **B:** Final snapshots of the orientation selective activation (*t* = 550 ms) for *RW*_ex_ = 0.25 with *β* = 0.9 (top, green circle in A) where the activation for certain orientations has destabilized and is spreading across the network and for *RW*_ex_ = 0.225, *β* = 0.5 (bottom, green star in A) where the activation is stable but still extends outside FFF, compare with Fig 7E. Panels on right are zoom-out of left. **C:** As A with the strength of inhibition increased by 50%, the contours determining the feasible operating range (green box as in Fig 7A–7C) were recomputed. **D:** As A with fixed *RW*_ex_ = 0.25 but varying inhibition strength C on the horizontal axis. The vertical slices at *RW*_ex_ = 0.25 in C and at *C* = −0.6 in D coincide.

The measures of the orientation selective component of the lateral spread of activation as studied in individual simulations in Figs 5 and 6 are now quantified over a range of *RW*_ex_ and *β*_rec_. The ranges of these parameters consistent with anatomical data is indicated in Figs 7–9 (between the white iso-*κ* contours reproduced from Fig 3). The agreement of model simulations with qualitative features from imaging data will be assessed in this and the following section. Fig 7A shows a map of normalized area (relative to the FFF area) of the selective activation, where each value is an average from simulations at 5 randomly selected map locations. Within the red contour (darker regions) the selective area is roughly equal to or smaller than the FFF of the stimulated region (e.g. Fig 7E, top). Outside this region the selective region is larger than the FFF (e.g. Fig 7D, top). Fig 7B shows a greyscale map of the proportion of the selective area with the correct orientation with respect to the underlying orientation preference map. Within the white contour (darker regions) the majority of the selective region has the correct orientation. For small *RW*_ex_ or small *β*_rec_ a significant proportion (more than 15%) of the selective activation is spurious (not in agreement with the preference map). Fig 7C shows a map of the ratio of the slopes (Naka-Rushton exponent) of the selective activation *n*_Sel_ and the general activation *n*_Act_. When this ratio *n*_Sel_/*n*_Act_ is large there is steeper radial decay of the selective activation (relative to the general activation) and a sharp transition to non-selectivity at the border of the FFF. Note that *n*_Act_ and *n*_Sel_ are independent of the choice of *η*_Sel_ and *η*_Act_ defined in (28).

In each case (Fig 7A–7C) there is a significant overlap between the regions with orientation selectivity confined to the FFF, with this selective activation having the correct orientation, with a characteristically steep decay of this region, and the anatomical range for the lateral connections in the model. In the green triangular region (overlayed in Fig 7A–7C), all of these characteristics are satisfied. We highlight that the operating range matching anatomy and functional data covers more than 20% of the (conservative) permissible range of *RW*_ex_ (0.1 < *RW*_ex_ < 0.55 = *RW*_in_).

The extent of the operating region for the model depends on values of the thresholds used in Fig 7A–7C. A value of 1.05 for normalized selective area is consistent with observations from [24], that the selective area is close to or marginally larger than the FFF area. A value of 85% for the proportion correct orientation was chosen heuristically, for smaller values isolated spurious selectivity occurs outside the FFF, e.g. compare Fig 7E and 7F. The value *n*_Sel_/*n*_Act_ = 1.3, also chosen heuristically, gives a sharp drop-off in the selectivity close to the FFF border. Reducing the threshold on the selective area, or the threshold on *n*_Sel_/*n*_Act_, by 10% would give a reduced by still existing operating region for the model, but increasing the proportion correct orientation threshold much beyond 85% rapidly reduces the operating region. Fig 8 illustrates the dependence of the operating region limits on each of the thresholds. The specific choice *η*_Sel_ = 0.5 could also affect the size of the model’s operating region for the normalised selective area. For example, increasing *η*_Sel_ could potentially increase the operating region for a fixed value of the threshold on the normalized selective area. However, the constraint on the ratio *n*_Sel_/*n*_Act_ (also providing an upper bound on the green region) would be unaffected as it is independent of the choice of *η*_*sel*_.

### Reduced inhibition leads to orientation selective activation outside stimulus footprint

Although an anatomical bias towards iso-orientation in the model’s connectivity profile was introduced through *β*_rec_, the bias is not necessarily evident at the population functional level (in the patterns of activity observed in simulations). This may originate from the non-linearities in the functional expression that combines excitatory and inhibitory activation. To further explore the role of the relative balance between E/I in this functional expression, we manipulate the strength of inhibition in the model (controlled by *C* in (16)). Recall that when *C* = 0 there is global E/I balance and for the reduced inhibition case here we reduce *C* toward 0 (from its standard value *C* = −0.4 to *C* = −0.2). Fig 9 (computed in the same way as Fig 7A) shows that with reduced inhibition, the region of the (*RW*_ex_, *β*_rec_)-plane with orientation selective activation confined to the FFF footprint (inside the red contour) is smaller. In general, maintaining the same values of other parameters, but reducing inhibition leads to a wider spread of orientation selective activation. Compare Fig 9B (bottom) where the stable pattern of activation extends outside the FFF with Fig 7B (bottom) where the activation is confined within the FFF. Furthermore, with strong orientation bias in the model’s lateral connections (large *β*_rec_), the spread of activation can destabilize and continue indefinitely, see Fig 9B (top). This activation outside the stimulated region has spurious orientation preference and is due to destabilized activity that spreads far beyond the boundary of the stimulated region. Note that this a distinct mechanism from the convergent excitation that generates activation with incorrect orientation when *RW*_ex_ is too small (see Fig 5B). For the case illustrated in Fig 9B (top), of the four stimulus presentations with *I*_0_, *I*_45_, *I*_90_ and *I*_135_, the condition *I*_0_ resulted in an continual spread of activation down and to the right of the FFF (blue-green regions), *I*_45_ above the FFF (purple regions), whilst *I*_90_ and *I*_135_ remained constrained to the FFF. These different responses across different orientations arise from a local imbalance in the preference map, which becomes exaggerated when input drives the model close to spatial instability, as is the case with reduced inhibition. With stronger inhibition, small imbalances in the spread of activity can occur across different orientations, but these don’t impact the validity of tuning of the response (e.g. see the slightly larger response to 135° in Fig 4B). We note that the destabilization is not contingent on there being local imbalance in the preference map, in general, moving in parameter space to the top left of Fig 7A, the unbounded activation would occur for all input orientations. In general, with reduced inhibition, there is no more overlap in the (*RW*_ex_, *β*_rec_)-plane between the anatomical operating range of the model (between the white contours) and the range where orientation selective activation is confined to the FFF.

With increased inhibition strength (*C* = −0.6) the operating region for the model increases slightly shifting to lower *RW*_ex_ and extending to larger *β*_rec_ values (Fig 9C). This illustrates how inhibition constrains the spread of activation to the FFF, even with larger orientation bias of lateral connections. Fixing *RW*_ex_ = 0.25, the operating region in terms of *C* and *β*_rec_ is illustrated in Fig 9D, with the largest extent in *β*_rec_ occurring at *C* = −0.4. For large values of *C* the model responses become predominantly feed-forward (input driven) and the profile of decay for the general and selective activation becomes similar (*n*_Sel_/*n*_Act_ tends to 1). As discussed above, without sufficient balance from inhibition activity can spread unbounded across cortex (top left region of Fig 9D).

## Discussion

This study reported on a neural field model of L2/3 of primary visual cortex, investigating the dynamics of stimulus driven, localized patterns of cortical activity. The aim was to understand the relationship between the anatomical properties of lateral connections and the functional propagation of cortical activation. The model featured a coarse-grained description of average membrane potential in 2D, with discrete sub-populations for different orientations. Orientation was encoded using a realistic orientation preference map that modulates inputs from LGN via L4 and/or the lateral connections within L2/3 sub-populations. Anatomical data quantifying the orientation bias of lateral connections was used to constrain model parameters [14].

VSDI experiments in V1 have shown that local, oriented visual stimuli elicit stable orientation-selective activation within the retinotopic footprint [24]. Recently two studies confirmed these findings, one using optogenetic stimulation [27] and another anatomofunctional study [15]. The cortical activation dynamically extends far beyond the retinotopic footprint, but the peripheral spread stays non-selective, which could be surprising given anatomical studies showing the orientation specificity of long-range connections [7,10,8,11,9]. However, this result could actually be expected given that (i) the quantified orientation bias is small (ii) decreases with distance [14] and (iii) holds only for a sub-part the functionally activated hyper-column (L4, pinwheel and interneuron cells do not show this bias [12, 65], but see [14, 15] for the pinwheel dependence).

Based on these anatomical studies, we designed a new connectivity function, flexibly parametrized to investigate clustering of connections, their orientation bias and balance between excitation and inhibition. We adopted the non-orientation specific nature of local excitatory connections [14] and inhibitory connections [16]. Taking motivation from [16], longer-range excitatory connections are proposed here to, although decaying with distance, form in rings at multiples of *Λ* (hypercolumn separation). This allows for the following important features to be captured: that excitatory connections 1) drop in number at a range *Λ*/2, 2) have a peak at a range *Λ* and 3) can extend several mm across cortex. Two parameters were tuned to agree with the available data from [14], the width of peaks in number of excitatory connections and their orientation bias (note we did not include an explicit representation of L4 nor a special connectivity rule at pinwheel locations). Similar results and conclusions would be expected if other parameters had been varied, e.g. those controlling the amplitude of excitatory peaks, or the width of inhibition.

We found a significant overlap between the anatomically relevant parameter range and patterns of cortical activation consistent with imaging experiments. Hence the imaging results can be reconciled with the reported level of orientation bias from anatomical studies. Specifically, [24] found a sharp decay of orientation selective activation at the stimulus retinotopic footprint border, resulting in peripheral activation that was not orientation selective. Our results demonstrate that this sharp decay is contingent on three factors: the diffuse clustering of long-range connections, the intermediate range (consistent with anatomy) of their orientation bias and the sufficient balance between excitation and inhibition. We note that although long-range connections are orientation biased, the recruitment at the target of a local network without orientation bias could result in a general non-orientation specific activation [27]. Furthermore, if the orientation bias of lateral connections is excessively strong, or if inhibition is particularly weak, the network operates close to an instability leading to unbounded cortical activation (a testable prediction, see below).

### Model predictions

Our modelling results predict a qualitative change in the spread of orientation selective activation for localized, oriented inputs if global inhibition strength is reduced. Under those conditions, we should observe selective activation outside of the retinotopic footprint, however, the exact orientation preference may not be preserved. Activation with spurious orientation preference is generated via spatial destablization of the localized activation generated by the input. These predictions could be addressed by manipulating the inhibitory cells pharmacologically [66–68] or optogenetically [27]. To confirm our model’s prediction though, one may have to go close to pathological epileptic conditions [69, 70]. Other approaches could be used by simply comparing anesthetized and awake conditions. Recently, [71] have indeed shown this to be a valuable approach for investigating E/I balance in the integrative properties of the cortical populations. Comparison of the dynamics of propagation of orientation-selective activity in awake or anesthetized conditions could hence provide the appropriate non-pathological test to probe our model’s prediction.

Spurious activation can also be generated by another mechanism. Activated regions inside the stimulated region can generate excitation at an equidistant range outside the stimulated region. The specific range is associated with the peak in the radial excitatory profile. This happens in the model when peaks in excitation are highly specific, in a parameter range that was ruled out from the operating regime of the model. This could be seen as an undesired consequence of the model’s design. However, the fact that, under particular circumstances, the preferred orientation of the horizontal propagation may be at odds with the underlying orientation preference map could unravel some new unexpected computational capacities of the horizontal network, which may be present in visual areas beyond V1/area 17. For instance, the ability to link information of position and orientation for non co-circular filters that could be of importance for processing objects with sharp angles. Inline with this hypothesis, [24] showed that the spread of orientation selective activity is not fixed but can increase when increasing spatial summation.

### Comparison with other models

The transition to an unbounded spread across the network, as contingent on a spatial modulation parameter (like *β*_rec_ controlling orientation bias in our model), has been observed in a one dimensional theoretical study of the neural field equation with purely excitatory connectivity [72]. A more common choice of connectivity function (e.g. difference of Gaussians) features a broader footprint for inhibition than for excitation [36, 37]. In our model excitation extends further than inhibition with additional peaks in excitation away from the origin [44]. The distance between excitation peaks fixes a regular length scale that stabilizes multi-bump patterns. In [41], without an explicit representation of orientation, localized inputs were shown to produce multiple bumps within a stimulated region. It was proposed that the connectivity’s excitatory peak separation could be equated with *Λ* (hypercolumn separation) and the spatial phase of multi-bump patterns governed by an orientation preference map. In the present work, we have shown that the localized patterns of orientation selective activation observed in [23] and [24] are well described by a superposition of these intrinsic multi-bump states.

Local models of orientation selectivity [32, 33] have been more widely studied than spatial models capturing interactions across columns. Spatial models of map development [55, 73, 46, 74, 57] do not focus on dynamics on sub-second timescales. Indeed, [74] does not consider dynamics or long-range connections within V1; see also commentaries in [75, 76]. The self-organizing map (SOM) model in [57] included, but did not show, the dynamics of stimuli responses. The focus of the study was on the long-timescale dynamics of map development, however, this class of model could be another candidate to investigate functional activation in the future. Integrate-and-fire neuron models of earlier thalamic and cortical processing stages proved successful for capturing the dynamics of orientation selectivity and tuning, but were restricted to a small patch of cortex without considering superficial layers accessible to imaging [77, 78]. [79] explored the role of patchy long-range connections in cortex in a general setting, whilst [80] looked at larger regions of cortex in an integrate-and-fire network, reporting complex spatio-temporal dynamics, but did not model orientation.

The neural field framework used here is an ideal level of description for comparison with VSDI recordings [38] that allowed us to simulate a large spatial domain extending beyond the local region of interest, thus avoiding issues with boundary effects. The four sub-population implementation used here was chosen because it allows for efficient computation of connectivity integrals using convolutions. In turn, this allowed for a comprehensive multi-parameter investigation of the model’s dynamics. Another choice would be to consider a single mixed population, where connection weights depend directly on orientation difference as has been considered in other spatial models [73, 81], including those posed in an elastic net framework [82, 83]. This would have the advantage of allowing direct implementation of the heterogeneity of long-range connections, however, the increased computational burden may not allow such a broad parameter investigation.

### Perspectives

[27] used optogenetic stimulation in combination with visual inputs and observed a non-orientation specific linear addition of the two inputs, which could be explored in our modelling framework. The model could also be used to investigate selective recruitment and spatial summation in regions between localized oriented stimuli. Two further properties were described in [24] that can be further investigated. First, as demonstrated with intracellular recordings, the lack of orientation selectivity observed at the mesoscopic level (VSDI) was due to a diversity of microscopic rules: some cells received untuned presynaptic input, but others a tuned presynaptic input with preferred orientation either agreeing with or different from the recorded cell. Such diversity in local cellular rules is to be linked to our observations that, with narrow excitatory peak widths or strong orientation bias (Fig 7B) or reduced inhibition (Fig 8), our model can easily produce spurious selective propagation. Adding diversity in the connectivity rules can thus easily lead to natural diversity in the tuning of the propagation. Second, increasing spatial summation increases the slope of selectivity decay at the stimulus boundary, whilst selective propagation reaches further across cortex; the model could also account for this property. More generally, the model could be used to make predictions to decipher the selective functional connectivity rules that link position and orientation in cortical space. For example, the model could be extended to differentiate inhibitory cell sub-classes as reported in [16]. As such it could generate functional predictions on e.g. the role of long-range basket cell connections that preferentially target cross orientations.

A lumped description of inhibition and excitation was used here with local cross-orientation inhibition. By separating out inhibitory and excitatory populations, one could consider different profiles for cross-orientation interactions including their spatial profile. This may change the model’s behavior and would be important if it is extended to consider inputs with multiple orientations that overlap spatially. Earlier stages of cortical and thalamic processing could be incorporated with differing properties of orientation tuning and bias of connections across V1 layers [84]. The four sub-population implementation used here can be viewed as a coarse discretization from a continuous representation of orientation, which could be considered in future work. For example, a recent paper studied spatio-temporal patterns with continuous orientation, but only on a 1D spatial domain [85]. Theoretical work characterising localized states in 2D space plus orientation would be an important first step. An extended feature space including spatial frequency (SF) could be used to investigate lateral connections in light of recent work showing interesting interactions between orientation and SF maps [86].

### Conclusion

Our model of orientation selectivity in V1 is the first of its kind, capturing the spatio-temporal dynamical spread of localized patterns of activation with a representation of orientation. Our study addressed an apparent conflict between the orientation bias of lateral excitatory connections in L2/3 of V1, as characterized in anatomical studies, and imaging studies on the lateral propagation of cortical activity for localized oriented visual stimuli. Simulations with the neural field model illustrated that observed levels of orientation bias in anatomical studies actually predict long-range activation outside of the retinotopic footprint of the stimulus, but with a sharply decaying profile of orientation selectivity, as observed in imaging studies. Without this sharp decay, which might occur with excessive orientation bias or diminished inhibition strength, the network could destabilize leading to unbounded spread of cortical activation.

## Supporting information

S1 Video Animations corresponding to Figs 5B, 5E and 7D, 7E, 7F are available as supporting information. (ZIP)

S1 Code Matlab source code for the model, allowing for independent reproduction of the results, is available as supporting information. The code is also available on the following public repository: https://github.com/QBME/rankin-chavane-neural-field/.
(ZIP)
